# Overall Polyp Detection Rate as a Surrogate Measure for Screening Efficacy Independent of Histopathology: Evidence from National Endoscopy Database

**DOI:** 10.3390/life14060654

**Published:** 2024-05-21

**Authors:** Mark Aloysius, Hemant Goyal, Tejas Nikumbh, Niraj Shah, Ganesh Aswath, Savio John, Amol Bapaye, Sushovan Guha, Nirav Thosani

**Affiliations:** 1 Division of Gastroenterology, Department of Internal Medicine, SUNY Upstate Medical University, Syracuse, NY 13210, USA; vamedicalresident@gmail.com (M.A.);; 2 Borland Groover-Downtown, Baptist Medical Center-Downtown, 836 Prudential Dr. Ste 801, Jacksonville, FL 32207, USA; 3 Department of Internal Medicine, The Wright Center for Graduate Medical Education, Scranton, PA 18510, USA; drtejasnikumbh@gmail.com; 4 Division of Digestive Diseases, Department of Medicine, The University of Missouri at Columbia, Columbia, MO 65211, USA; 5 Shivanand Desai Center for Digestive Disorders, Deenanath Mangeshkar Hospital and Research Center, Pune 411004, India; 6 Section of Endoluminal Surgery and Interventional Gastroenterology, McGovern Medical School and UT Health Science Center, UTHealth Houston 6431 Fannin St, MSB 4.020, Houston, TX 77030, USA

**Keywords:** screening colonoscopy, adenoma detection rate, polyp detection rate, colon cancer, screening

## Abstract

Adenoma detection rate (ADR) is challenging to measure, given its dependency on pathology reporting. Polyp detection rate (PDR) (percentage of screening colonoscopies detecting a polyp) is a proposed alternative to overcome this issue. Overall PDR from all colonoscopies is a relatively novel concept, with no large-scale studies comparing overall PDR with screening-only PDR. The aim of the study was to compare PDR from screening, surveillance, and diagnostic indications with overall PDR and evaluate any correlation between individual endoscopist PDR by indication to determine if overall PDR can be a valuable surrogate for screening PDR. Our study analyzed a prospectively collected national endoscopy database maintained by the National Institute of Health from 2009 to 2014. Out of 354,505 colonoscopies performed between 2009–2014, 298,920 (*n* = 110,794 average-risk screening, *n* = 83,556 average-risk surveillance, *n* = 104,770 diagnostic) met inclusion criteria. The median screening PDR was 25.45 (IQR 13.15–39.60), comparable with the median overall PDR of 24.01 (IQR 11.46–35.86, *p* = 0.21). Median surveillance PDR was higher at 33.73 (IQR 16.92–47.01), and median diagnostic PDR was lower at 19.35 (IQR 9.66–29.17), compared with median overall PDR 24.01 (IQR 11.46–35.86; *p* < 0.01). The overall PDR showed excellent concordance with screening, surveillance, and diagnostic PDR (r > 0.85, *p* < 0.01, 2-tailed). The overall PDR is a reliable and pragmatic surrogate for screening PDR and can be measured in real time, irrespective of colonoscopy indication.

## 1. Introduction

Poor-quality colonoscopy increases the incidence and mortality related to interval colorectal cancers (CRC) [1]. The quality of colonoscopy varies widely among endoscopists; hence, there are established quality standards for colonoscopy such as adequacy of bowel preparation, cecal intubation rates, and withdrawal time [2]. In addition, the adenoma detection rate (ADR) is the proportion of colonoscopies with at least one adenoma or adenocarcinoma detected compared to the total number of colonoscopies performed. It is widely considered the most important quality measure for colonoscopy and was first proposed by the Multi-Society Task Force in 2002 [3,4,5]. Later, the American College of Gastroenterology (ACG) and the American Society of Gastrointestinal Endoscopy (ASGE) changed the ADR definition to include screening-only colonoscopy in ADR in 2006. 

ADR is currently considered the “gold standard” quality indicator for screening colonoscopy [4,5,6]. The ADR of an endoscopist is negatively associated with the patient’s post-colonoscopy CRC risk, with each 1% rise in ADR resulting in a 3% reduction in the risk of CRC [1,7,8]. The ADR benchmark is 25% overall, with 30% in men and 20% in women, and it has increased over time, reaching 39% in the USA [7]. Although ADR is the most validated quality indicator, it is neither perfect nor sufficient due to its many limitations [8]. ADR is cumbersome to measure as it is dependent on pathology reporting time. Moreover, sessile-serrated polyps are not included in ADR measurements. Also, efficiently measuring ADR might be difficult because many colonoscopies are performed for reasons other than screening. Proportions of screening procedures also vary by the endoscopist. 

PDR (polyp detection rate) is defined as the proportion of procedures in which at least one polyp was detected over the total number of colonoscopies [9]. PDR is an immediate measure, regardless of pathology results. has been studied as it is easily automated from endoscopy reports. Studies have shown that polyp detection can parallel adenoma detection and may confer CRC protection [10]. ADRs of 25% and 15% for men and women, respectively, have been found to correlate with polyp detection rates of 40% and 30% [11]. PDR is a strong proxy candidate for ADR. However, PDR has not been validated in prospective studies and is thought to be easily manipulated by endoscopists. Currently, there is no consensus regarding the equivalence of overall-ADR and individual ADR for different indications. A recent retrospective study showed the equivalency of overall-ADR (polyps detected from screening, surveillance, and diagnostic indications) to be comparable with screening-ADR, but it awaits confirmation from large-scale retrospective registry and prospective studies [11]. Another retrospective analysis showed the prevalence of polyps and adenomas differs based on colonoscopy indication, and distinct ADR and PDR targets may need to be established for different colonoscopy indications [12]. Also, a recent study of over 100 colonoscopies confirmed an excellent correlation between PDR and ADR [13].

We hypothesized that the overall PDR from all colonoscopies could parallel and be a surrogate for individual PDR for screening, surveillance, and diagnostic indications. Therefore, we aim to correlate individual endoscopist PDR with the indication to determine if overall PDR can be a valuable surrogate for screening PDR.

## 2. Materials and Methods

### 2.1. Study Design

The National Institutes of Health (NIH) Clinical Outcomes Research Initiative (CORI) database is a large-scale repository from a US multicenter consortium of 108 sites from 87 practices. It is a clinical database that collects data on gastrointestinal endoscopic procedures, including colonoscopies, upper endoscopies (esophagogastroduodenoscopies), and other related procedures. The CORI was created as a means of studying gastrointestinal (GI) endoscopy-related utilization and outcomes in diverse clinical practice settings, including private, community, academic, governmental, and health maintenance organizations (HMOs). All participating sites use a computerized report system to generate GI procedure reports, which are deposited electronically to a central data repository after quality testing and compiled into the National Endoscopy Database (NED). The NED contains data collected by the Clinical Outcomes Research Initiative (CORI) over a 15-year period, from 2000 to 2014 [14,15]. Anonymized data are collected and stored per strict HIPAA standards, and users must obtain data-user agreements and institutional review board (IRB) approval. This study was approved by the Wright Center of Graduate Medical Education IRB [1692454-2].

We queried version 4 of the CORI-NED database (CORI-V4), which includes data recorded from 2009 to 2014 undergoing colonoscopy. A total of 354,506 colonoscopies were analyzed in patients >18 years of age. Procedures were stratified based on the endoscopist’s volumes into quartiles. Colonoscopies performed by endoscopists with a low colonoscopy volume (performing <50 cases per year) were excluded since there is an association between physician ADR and post-colonoscopy colorectal cancer [16]. Colonoscopies with incomplete procedure-related data, inadequate bowel preparation, inability to visualize polyps of at least 5 mm, incomplete cecal intubation, and withdrawal time < 6 min were also excluded, as these are key components of high-quality colonoscopy [2]. Separate data on IBD was not identifiable in the NED database. 

### 2.2. Study Cohort

#### Definitions

PDR (percentage of colonoscopies in which one or more polyps were detected) was studied for each endoscopist. We defined the following PDR categories:

(1) Screening—PDR as the number of screening procedures where 1 or more polyps were detected divided by the total number of screening colonoscopies in average-risk patients >50 years of age; The screening group did not have prior FOBT.
(2) Surveillance—PDR as the proportion of surveillance colonoscopies in which at least 1 polyp was found;
(3) Diagnostic—PDR as the proportion of diagnostic colonoscopies in which at least 1 polyp was found;
(4) Overall—PDR as the number of procedures where 1 or more polyps were detected over the total number of colonoscopies (irrespective of indication).

In addition, we evaluated the colonoscopies where the histopathology reports were available. We calculated the ratio of adenomas (serrated, tubular, villous, or tubulo-villous adenomas) to all polyps (including hyperplastic, inflammatory polyps, or benign colonic mucosa) as the adenoma-to-polyp ratio. 

### 2.3. Statistical Analysis

We analyzed the data obtained from various colonoscopy indications and calculated polyp detection rates (PDR). Analysis of Variance (ANOVA) was utilized to compare the separate groups of colonoscopy indications (independent variable) with PDR (dependent variable), allowing us to assess any significant differences in PDR across different indications. To evaluate the concordance between various factors, data were meticulously paired, ensuring appropriate matching between relevant variables. Concordance analysis was then performed, used to assess the agreement or similarity between two sets of data. 

In our study, the Spearman rank correlation coefficient was chosen for its suitability in assessing non-linear relationships and non-normally distributed data. To ascertain the normality of the data, skewness and kurtosis were calculated, revealing a non-parametric distribution. Consequently, given the non-parametric nature of the data, Spearman’s rank coefficient was employed to assess correlations, providing insights into the strength and direction of association between variables. Furthermore, a 95% confidence interval was utilized to quantify the precision of the estimated correlation coefficients, with statistical significance set at *p* < 0.05, ensuring robustness in our findings.

We also conducted a size sensitivity analysis using receiver operating characteristics (ROC) to determine the optimal size threshold for predicting the diagnosis of an adenoma versus a benign lesion. We plotted the true positive rate (sensitivity) against the false positive rate (1-specificity) for various polyp size thresholds. The area under the curve (AUC) in the ROC analysis was evaluated to determine the optimal threshold, with higher AUC values indicating better discriminatory ability. The size threshold corresponding to the highest AUC was identified as the optimal cutoff point for distinguishing adenomas from benign lesions. 

IBM SPSS version 28.0 was used for data analysis. We employed granular analyses by quartiles and polyp sizes to explore the relationships between variables in greater detail. We divided the data into quartiles, allowing us to examine how different segments of the dataset contributed to correlations. 

## 3. Results

A total of 354,505 colonoscopies were initially screened for eligibility. Approximately 55,585 colonoscopies were excluded because of incomplete cecal intubation, inadequate bowel preparation, withdrawal time <6 min, or because they were performed by providers with <50 recorded procedures. A total of 298,920 colonoscopies that met inclusion criteria (*n* = 110,794 (37%) average-risk screening, *n* = 83,356 (28%) average-risk surveillance, *n* = 104,770 (35%) diagnostic) were performed by 421 endoscopists at 83 sites between 2009 and 2014. The female-to-male ratio was 0.48:0.51. Overall median PDR for females and males was 25.49 and 36.95, respectively. 

**Figure 1:**
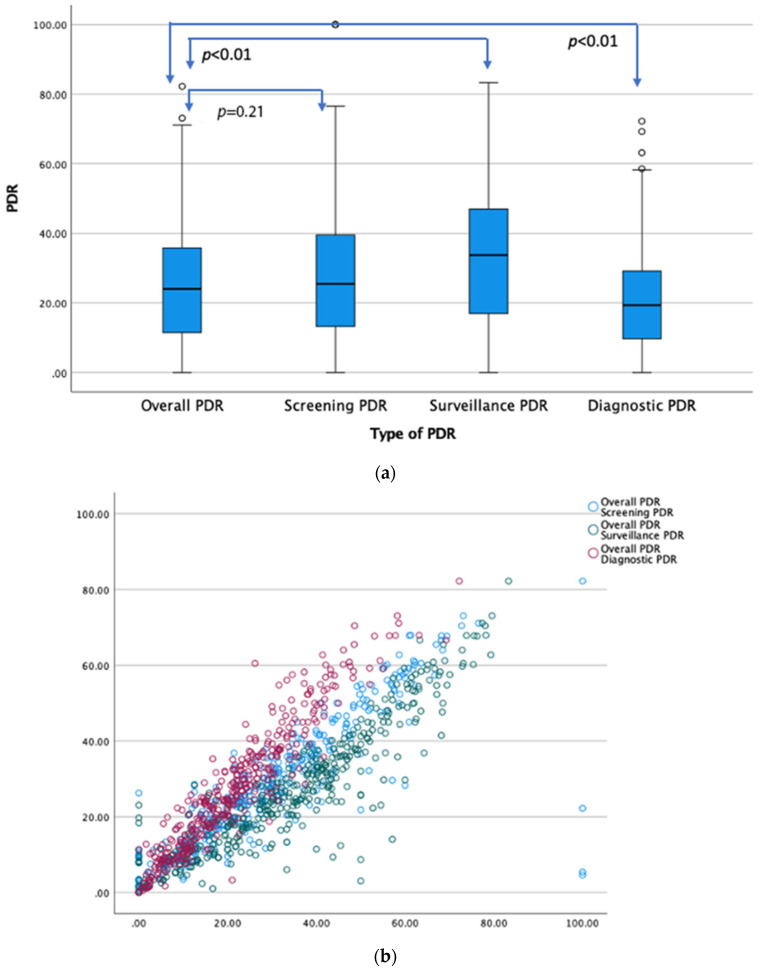
(**a**) Stem and leaf plot (median and IQR) comparing distribution of overall PDR with screening, surveillance, and diagnostic PDRs, respectively. (**b**) Overlay scatter plot showing correlation between overall PDR with screening (r = 0.858, *p* = 0.01), surveillance (r = 0.9299, *p* = 0.001), and diagnostic PDRs (r = 0.952, *p* = 0.01), respectively. Each circle represents the intersecting correlate of these variables for a given colonoscopist. Correlations were highly significant at *p* = 0.01 (2-tailed).

The median screening PDR was 25.45 (IQR 13.15–39.60), comparable to the median overall PDR of 24.01 (IQR 11.46–35.86, *p* = 0.21). Median surveillance PDR was higher at 33.73, (IQR 16.92–47.01), and median diagnostic PDR was lower at 19.35 (IQR 9.66–29.17), compared with median overall PDR 24.01 (IQR 11.46–35.86; *p* < 0.01). The overall PDR showed excellent concordance with screening, surveillance, and diagnostic PDR (r > 0.85, *p* = 0.01, 2-tailed) (Figure 1a,b). 

Further, we analyzed the data for each of the four groups, i.e., screening, surveillance, and diagnostic colonoscopy, and the total number of colonoscopies, by stratifying each group into quartiles. In each group, the granular data analysis by quartiles (Supplementary Figures S1–S4) showed excellent concordance with screening-, surveillance-, diagnostic-, and overall PDR, irrespective of the procedural volumes of the endoscopist. 

Histopathology was available for 36% of polyps (*n* = 33,948). On analyzing the polyps by size, 85% of the polyps were sub-centimeter, while 15% were ≥10 mm in size. The proportion of polyps removed by size (mm) did not vary by indication (Supplementary Table S1). The histology of polyps was comparable when divided into left-sided and right-sided colonic lesions (Supplementary Table S2). The mean adenoma-to-polyp ratio was similar for right-sided as well as left-sided lesions (Supplementary Figure S5).

**Figure 2:**
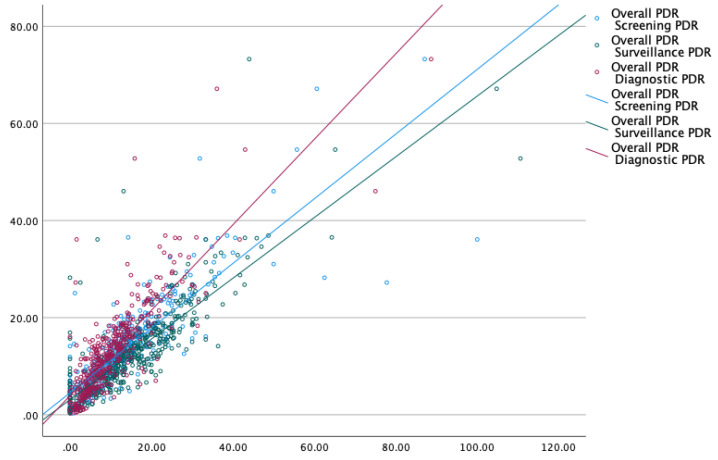
Polyp detection rate after excluding polyps <5 mm.

**Figure 3:**
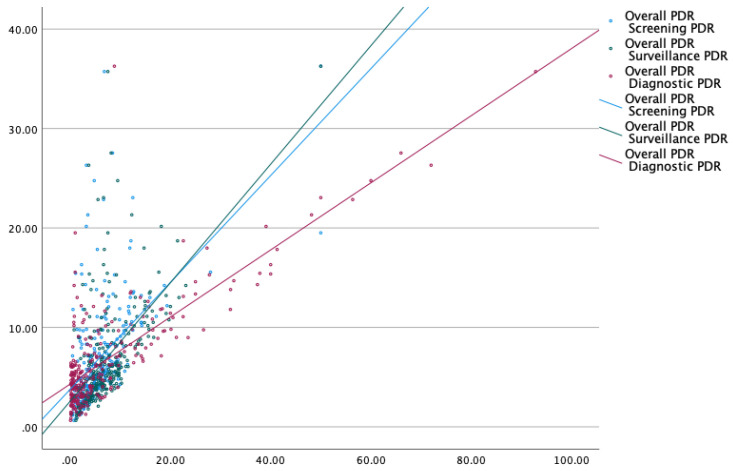
Polyp detection rate after excluding polyps <10 mm.

**Figure 4:**
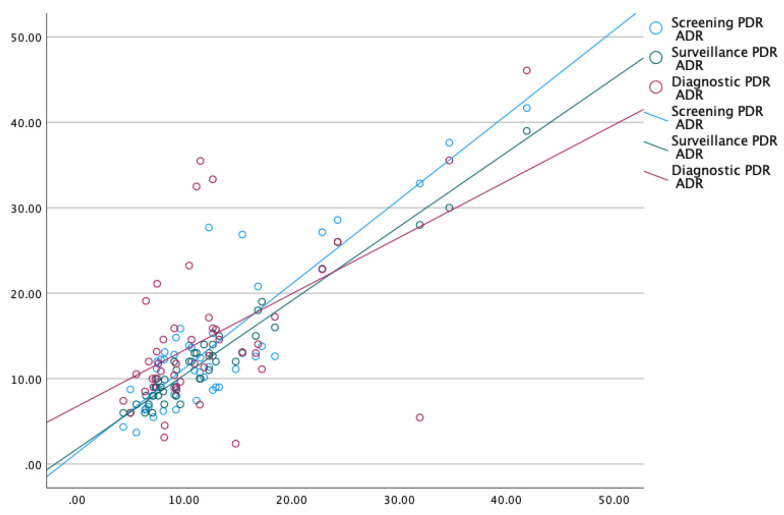
Correlation of ADR with overall PDR, screening PDR, surveillance PDR, and diagnostic PDR (*n* = 46).

The hyperplastic polyp is the most common non-neoplastic polyp in the colon. Most are small and less than 5 mm in size. In contrast, adenomas are neoplastic polyps that account for most larger polyps (larger than 1 cm) [10,11]. Due to the unavailability of histological data for 2/3rd of polyps, we performed two subgroup analyses excluding polyps ≤5 mm and ≤10 mm with the intent of excluding hyperplastic polyps. Our findings were valid even after excluding diminutive polyps <5 mm, strengthening the validity of this approach (Figure 2 and Supplementary Table S3). The results were strikingly similar to the analysis using polyps of all sizes, as overall PDR correlated well with screening PDR (*p* < 0.01), surveillance PDR (*p* < 0.01), and diagnostic PDR (*p* < 0.01), respectively (Figure 3 and Supplementary Table S4). For the small number of polyps whose histology was available as a complete set for a fraction of endoscopists (*n* = 46), ADR was calculated for screening colonoscopies and compared with their screening ADR, surveillance ADR, and diagnostic ADR (Figure 4 and Supplementary Table S1). We analyzed a subset of colonoscopies where complete histological data were available for 46 endoscopists. ADR demonstrated excellent correlation with overall PDR, screening PDR, and surveillance PDR, and good correlation with diagnostic PDR.

**Figure 5:**
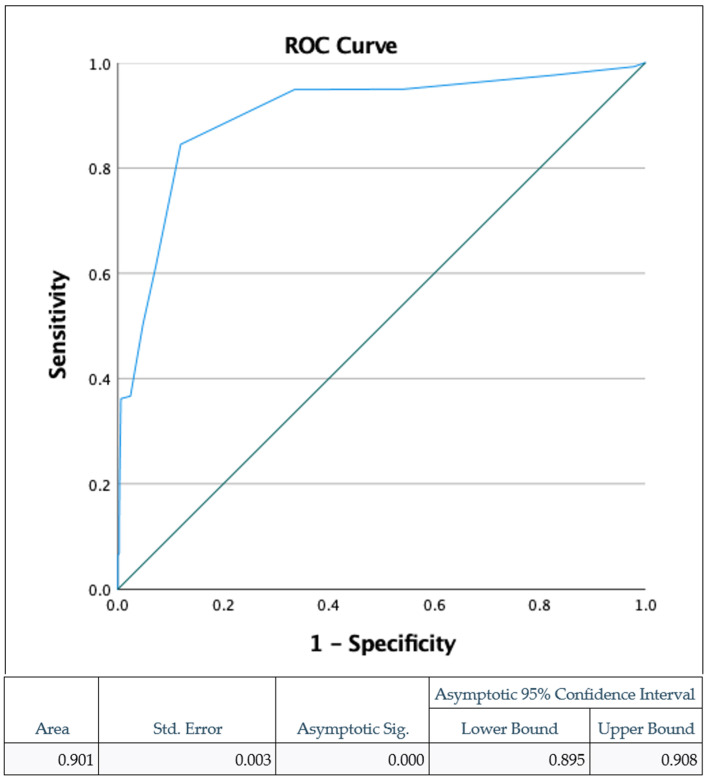
Receiver operating curve (ROC) characteristics of adenoma detection based on polyp size threshold at >5.5 mm with a sensitivity of 85% and false positive rate of 11%. Area under curve (AUC)—0.901.

We also performed a size sensitivity analysis using receiver operating characteristics (ROC) to determine the size threshold of the study, which predicted the diagnosis of an adenoma versus a benign lesion (Figure 5). A polyp size larger than a threshold of 5.5 mm predicted adenoma detection with a good sensitivity of 84.5% and a low false positivity rate of 11%.

## 4. Discussion

Overall PDR appears to be a valuable and pragmatic quality metric for assessing colonoscopy quality, showing excellent concordance with screening and can be measured in real time, irrespective of the procedure indication. Our study is the largest in terms of the number of colonoscopies, demonstrating that overall PDR is a feasible colonoscopy quality metric with proven equivalency with screening PDR in the absence or unavailability of histology and ADR reporting.

Quality metrics in screening colonoscopy are measured to prevent interval CRC. Moreover, it has been shown that continuous audit of colonoscopies has helped improve the detection of polyps [17]. Moreover, there has been a significant upward trend in the detection of sporadic malignant polyps, especially in the young onset CRC age group of 40–49 years [18]. While ADR is a robust measure, it is also cumbersome. ADR reporting requires histopathological confirmation of adenoma [19]. It is also essential to have a linkage in the same system that has the endo-writer software [20]. This seamless integration appears to be a widespread challenge in health systems. Often, these reports must be manually entered or scanned into the electronic health record, making it a cumbersome practice [11,21]. Moreover, ADR does not account for serrated polyps, increasingly recognized as precursors for CRC development [22,23]. Many colonoscopies are performed for non-screening purposes (surveillance or diagnostic), limiting the number of examinations eligible for ADR assessment [12]. 

Bowel preparation is a key quality indicator in colonoscopy, with the US Multi-Society Task Force recommending ≥85% of outpatient examinations have an adequate preparation [24]. However, there are studies showing that there is no difference in ADR between the optimal vs fair grades of bowel preparation [25,26,27]. In fact, PDR and ADR decreased at the highest levels of bowel cleanliness [28]. While we excluded colonoscopies with inadequate bowel preparation, bowel preparation has a clear effect on the time of entry, the duration of the procedure, the discomfort of the patient during the procedure, and efforts to improve the quality of bowel.

Recent discussions have focused on two potential ways to increase the ADR despite poor endoscopic performance. First is “indication gaming”, in which a patient who comes with symptoms is eligible for screening and an endoscopist retrospectively labels a colonoscopy as a non-screening colonoscopy if an adenoma is not discovered [29]. Second is the “one and done” phenomenon, where the endoscopist removes one adenoma and does not check the rest of the colon thoroughly since he has already fulfilled the metric [6,30]. Calculating ADR for individual endoscopists cannot distinguish between those who find only one versus more than one adenoma per colonoscopy [31].

The outcome quality of screening colonoscopies is mainly influenced by individual colonoscopist factors [32]. Previous studies have shown that large sample sizes (e.g., 500) are required for a reliable assessment of an endoscopist’s ADR [33]. Serrated polyp detection is linked to more recent training completion and higher procedure volume [34]. There is an association between ADR and serrated polyp detection, with endoscopists with a higher ADR detecting significantly more serrated lesions than those with a lower ADR [35]. Colonoscopy procedural volume increases ADR and PDR in gastroenterology trainees with at least 275 colonoscopies to achieve competence [36]. However, a ratio of ADR to PDR (adenoma detection quotient) had an excellent correlation with ADR only when >177 procedures per endoscopist were evaluated [37]. 

Our study demonstrates that overall PDR correlation with screening, diagnostic, and surveillance PDRs does not change for endoscopists, irrespective of their volume quartile for overall PDR and individual PDRs as per the indication. Our study validates the findings from a previous meta-analysis of the association between endoscopist annual procedure volume and colonoscopy quality, which found that higher annual colonoscopy volumes did not correlate with ADR [38]. Interestingly, this study found that a higher annual colonoscopy volume correlated with a higher cecal intubation rate but not with ADR or post colonoscopy CRC rate. The adenoma to polyp ratio has been shown to be 0.70 in previous studies, which is similar to our result [25,26,32] [39]. A multi-national meta-analysis of 31,623 colonoscopies from 25 studies across nine countries found that a conversion factor of 0.688 can be used to calculate ADR from PDR [40]. About 55% of the polyps in our study were less than 5 mm in size, and the PDR to ADR prediction was approximately 2:1. Similar findings were also reported by another study from CORI, further validating our findings [41].

PDR offers real-time data during the colonoscopy as compared to ADR. There is no need for histological confirmation, making the reporting process more manageable. It is important to note that the polyp yield is impacted by the withdrawal time [42,43]. PDR has previously shown a strong positive correlation with ADR [11,21,37,41]. PDR also correlates strongly with ADR among trainee endoscopists [44]. Most endoscopists perform colonoscopies on patients for a variety of indications. A previous study by Kaltenbach et al. showed that extending ADR to encompass surveillance and diagnostic exams may also encourage endoscopists to enhance quality for those purposes [11]. Our results are in concordance, showing a positive correlation between overall PDR and PDR, irrespective of the indication. 

Many previous studies have concluded that overall-ADR has a strong positive correlation with ADR for screening colonoscopies and is an accurate surrogate for ADR [37,45]. The overall-ADR may be a better colonoscopy quality indicator as it simplifies measurement and may prevent gaming of the ADR metric by changing the colonoscopy indication [11,29,46]. Similarly, overall PDR has strong concordance with screening PDR. In a single-center retrospective study of 13,054 colonoscopies, the PDR was highest in colonoscopies prompted by a positive FIT test. The overall PDR correlated with the screening PDR (22.1% vs. 20.4%; *p* = 0.15) [47]. In another study, screening PDR was compared with PDR for polyps ≥5 mm and found there was no difference in the PDR despite the indication [48]. Moreover, reporting overall PDR rather than screening PDR would simplify PDR measurement and eliminate the potential for gaming the PDR by changing the colonoscopy indication.

The strength of our study is that a large sample of real-world data was utilized. The CORI-NED database has been analyzed to assess endoscopic practice patterns, treatments, and management, while also aiding in research hypothesis development and quality measure reporting. CORI has strict quality-control measures for all its data. The data repository is checked for anomalies daily, and unusual activity prompts contact by CORI staff [15,49]. Moreover, the data are representative of various gastroenterology practice settings, with most sites being community-based, followed by veterans’ administration and academic hospitals.

Our study results also have limitations, most of which are inherent to large database studies. First, this is a retrospective study of a database. Second, the study is prone to site-selection bias [50]. Around 15 million colonoscopies are performed in the United States annually [51]. The sites that chose not to participate in CORI-NED may differ in their clinical practice from the participating sites. However, strict quality control, a real-time data repository, multisite involvement, and a large number of procedures strengthen this database, providing real-world data on endoscopy practice. Although PDR is readily available as a colonoscopy metric, it has the disadvantage of including hyperplastic polyps, which may be erroneously used as a surrogate for ADR. In real-world scenarios of ADR reporting, histological confirmation of resected polyps is not linked efficiently and in a timely manner, as there is a delay in processing immunohistochemistry. One of the drawbacks of our study was the incomplete nature of histology reporting. Despite the unavailability of histology for the polyps of most endoscopists, we analyzed a subset of colonoscopies where complete histological data were available for 46 endoscopists. Here, ADR demonstrated excellent correlation with overall PDR, screening PDR, and surveillance PDR, as well as diagnostic PDR. 

Screening colonoscopy has allowed us to make huge strides in decreasing the incidence and mortality of CRC. As we go forward, we need to continue implementing quality improvement programs that focus on enhancing various aspects of colonoscopy, of which metrics such as ADR are the most studied and objective parameters. The role of artificial intelligence (AI) in increasing polyp detection rates in colonoscopy has emerged as a promising area of research and clinical application. AI technologies, particularly deep learning algorithms, can analyze endoscopic images and videos to assist endoscopists in real-time polyp detection, characterization, and classification. Several studies have demonstrated the potential of AI for increasing polyp detection rates. For example, a study reported by Wang et al. demonstrated an AI system capable of real-time polyp detection during colonoscopy, achieving a sensitivity of 94.4% and a specificity of 95.9% [52]. Similarly, Byrne et al. evaluated an AI system for polyp detection and found that it significantly increased adenoma detection rates compared to standard colonoscopy [53]. Moreover, AI technologies continue to evolve and improve, with ongoing research focused on enhancing their performance, generalizability, and integration into clinical practice. As AI algorithms become more sophisticated and widely adopted, they have the potential to revolutionize colorectal cancer screening and surveillance by improving polyp detection rates, reducing miss rates, and ultimately improving outcomes.

These results are the most extensive real-world data available to date from an NIH-maintained registry and can have significant clinical practice implications. Simplifying polyp detection reporting methods will improve compliance. Overall PDR is immune to indication gaming. It is easy and practical to measure PDR in real-time without delay for histology reporting compared with ADR. Gastroenterologists and their practices are likely to favor this quality metric as it is independent of “screening only” colonoscopies, procedural volume, and histology confirmation. Our findings are not meant to replace ADR as a quality metric for colonoscopy, which is a well-validated predictor of post-colonoscopy CRC, but as an alternative quality metric that is better than no reporting when the histopathology of resected polyps is unavailable. This is even more significant when the incentive to report ADRs was withdrawn by the Center for Medicare Services (CMS) in 2019. Subsequently, all 3 GI societies (AGA, ASGE, and ACG) responded in a concerted effort to reinstate ADR as part of MIPS (merit-based incentive payer system) by CMS. Regardless of the requirement by CMS, all gastroenterologists must monitor the quality of colonoscopies using a metric. The utilization of report cards and having an extra observer during colonoscopy to detect polyps have been linked to enhanced ADRs and are recommended for potential adoption in endoscopy centers [54].

## 5. Conclusions

We were able to show that overall PDR shows excellent concordance with all 3 PDRs (screening, surveillance, and diagnostic), independent of the procedural volume of the endoscopists. Furthermore, there is no difference in adenoma to polyp ratio between the proximal and distal colon, which was within a narrow range for all 421 endoscopists in the study. Overall PDR is unaffected by the proportion of screening colonoscopies performed by the colonoscopist. Thus, PDR as a simple and easy-to-calculate quality metric is promising and should be further evaluated in different practice settings. Further studies from large endoscopy databases, clinical trials, and meta-analyses will be required to establish overall PDR as a key quality metric for colonoscopy independent of histopathology.

## Data Availability

NIDDK Repository Acknowledgement. The CORI Investigators conducted the CORI study and were supported by the National Institute of Diabetes and Digestive and Kidney Diseases (NIDDK). The data from the CORI study reported here were supplied by the NIDDK Central Repository. This manuscript does not necessarily reflect the opinions or views of the NIDDK Central Repository or the NIDDK.

## Supplementary Materials

The following supporting information can be downloaded at: https://www.mdpi.com/article/10.3390/life14060654/s1, Figure S1: Granular data analysis of all colonoscopies by volume quartiles. Figure S2: Granular data analysis of all screening colonoscopies by volume quartiles. Figure S3: Granular data analysis of all surveillance colonoscopies by volume quartiles. Figure S4: Granular data analysis of all diagnostic colonoscopies by volume quartiles. Figure S5: The mean adenoma-to-polyp ratio for left-sided vs. right-sided colon. Table S1: Proportion of polyps removed by size (mm) did not vary by indication. Table S2: Histopathology was available for 36% of polyps (*n* = 33,948).
