# Supplementary material for: Overall Polyp Detection Rate as a Surrogate Measure for Screening Efficacy Independent of Histopathology: Evidence from National Endoscopy Database
Source: Life (Basel). 2024 May 21;14(6):654. doi: 10.3390/life14060654 (PMC11204558; doi:10.3390/life14060654)
Supplement: Supplementary file 1 [file life-14-00654-s001.zip › life-2886338-supplementary.pdf]

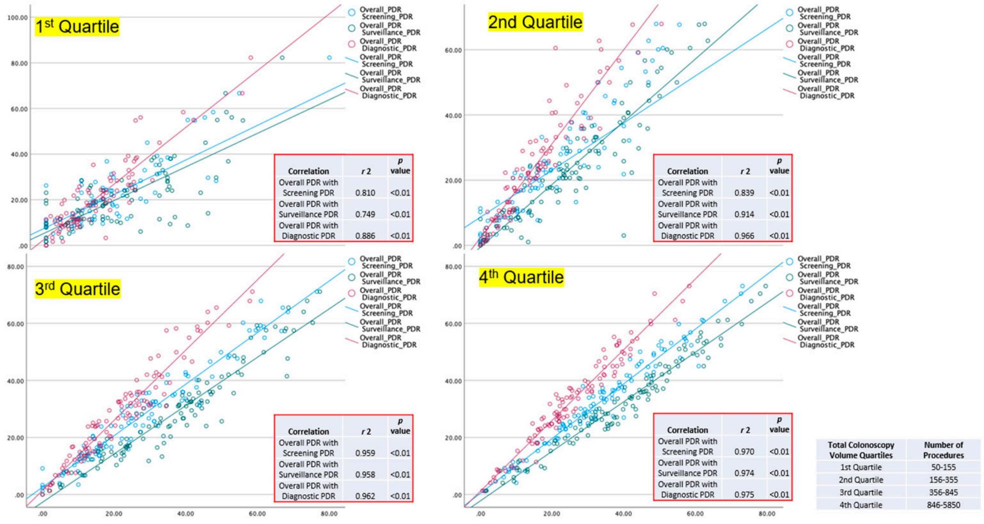

Supplementary Figure S1, Granular data analysis of all colonoscopies by volume quartiles

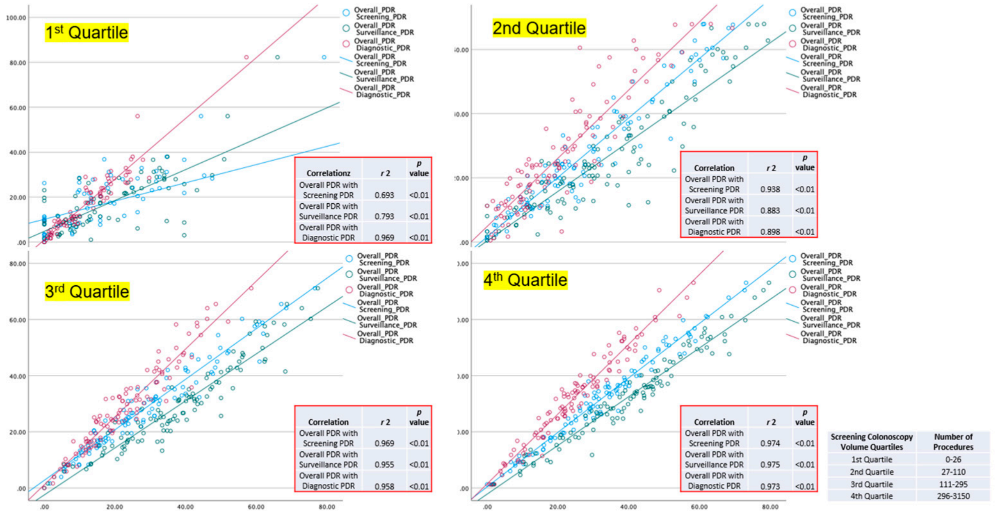

Supplementary Figure S2: Granular data analysis of all screening colonoscopies by volume quartiles

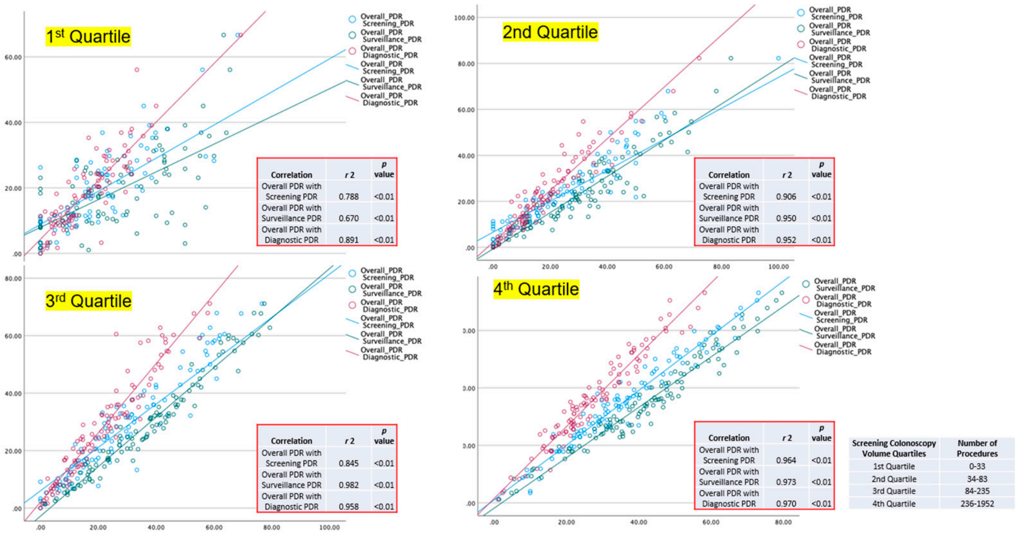

Supplementary Figure S3: Granular data analysis of all surveillance colonoscopies by volume quartiles.

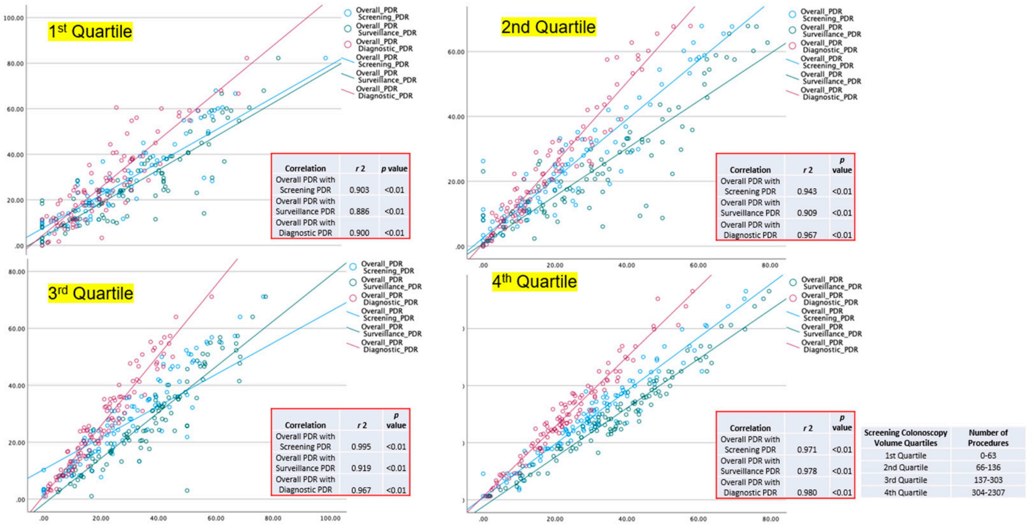

Supplementary Figure S4: Granular data analysis of all diagnostic colonoscopies by volume quartiles

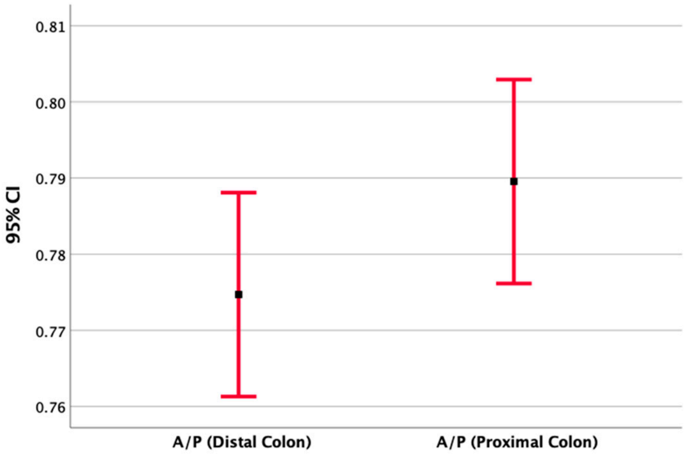

Supplementary Figure S5: The mean adenoma-to-polyp ratio for left-sided vs. right-sided colon;

	Polyps by Size n (%)				
Indication	>15 mm	10-15 mm	5-9 mm	0-5 mm	Total
Diagnostic	986 (4)	3141(13)	6436 (28)	12768 (55)	23331(100)
Average risk screening	994 (3)	4129 (12)	9867 (28)	20368 (58)	35358 (100)
Average risk surveillance	1150 (3)	3908 (11)	9086 (26)	21021(60)	35165 (100)
Overall	3130 (3)	11178 (12)	25389 (27)	54157 (58)	93854 (100)

Table S1: Proportion of polyps removed by size (mm) did not vary by indication

Polyp pathology	Proximal to splenic flexure	Distal to splenic flexure
Benign colonic mucosa	395	406
Hyperplastic	2961	3042
Inflammatory	292	306
Serrated	906	879
Tubular	11302	11561
Tubulovillous or Villous	964	934
**Total polyps**	**16820**	**17128**

Table S2: Histopathology was available for 36% of polyps (n=33,948)
